# Novel Dithiocarbamic Flavanones with Antioxidant Properties—A Structure–Activity Relationship Study

**DOI:** 10.3390/ijms252413698

**Published:** 2024-12-21

**Authors:** Mihail Lucian Birsa, Laura Gabriela Sarbu

**Affiliations:** Department of Chemistry, Alexandru Ioan Cuza University of Iasi, 11 Carol I Blvd., 700506 Iasi, Romania

**Keywords:** antioxidants, benzopyrans, dithiocarbamates, flavanones, radical enolates, benzylic radical

## Abstract

The antioxidant properties of some 3-dithiocarbamic flavanones were investigated. Based on a previous study, we selected three frameworks that proved to be the most active ones. By varying the nature of the substituent at the para-position of flavanone ring **B**, a structure–activity relationship study on radical scavenging activities was performed. The influence of these substituents (H, F, Cl, Br and I) was evaluated in relation to DPPH, ABTS and FRAP. The results indicated that the presence of the halogen substituent induced better antioxidant properties than ascorbic acid and BHT. The radical scavenging activities were found to decrease in the following order: F > Cl > Br > I > H. This is correlated with the decrease in electronegativity and withdrawing inductive effect of these substituents, which make the C(2)-H bond of the benzopyran ring prone to hydrogen radical transfer.

## 1. Introduction

Reactive oxygen species (ROS) are derivatives of molecular oxygen, which display a low stability and high reactivity. In humans, both radical and non-radical ROS are mainly generated during the normal cell metabolism by various enzymes and mitochondrial processes [1,2]. There is a fine balance between the formation and removal of ROS, since these processes are important in various biological systems, but at the same time, the exceeding of their levels must be prevented in order to avoid reaching cytotoxic concentrations [3,4,5,6]. Although the cells present enzymatic-based antioxidant defense mechanisms, there are a series of non-enzymatically processes acting as antioxidants in compounds that are endogenously synthesized [7]. Among these, there are antioxidants that are acquired through dietary sources [8,9,10,11].

From this point of view, flavonoids—a large group of naturally occurring heterocyclic organic compounds found in fruits, vegetable, tea and wine—are potentially good candidates. Flavonoids have a great diversity in chemical and biochemical properties that provide them with biological activity [12,13,14,15,16,17,18,19,20]. With the multitude of substitution patterns on the C_6_-C_3_-C_6_ backbone, more than 13,000 flavonoids are known [21]. The attention that they receive is a direct consequence of the many biological activities that this class of compounds displays. It is to be mentioned that for centuries, mixtures that contain flavonoids have been used in traditional medicine for the treatment and prevention of various infectious and toxin-mediated diseases, such as wound infections, acne, respiratory infection, gastrointestinal disease and urinary tract infections. These compounds are also known to be good antioxidants [22,23,24,25,26]. Cancer or cardiovascular diseases are believed to be protected by this mechanism of action of flavonoids [27]. Several mechanisms of the antioxidant capacity of flavonoids have been reviewed [28,29,30].

It is also worth noting that although antioxidant treatments have been used for a long time to manage acute or chronic diseases, small molecule-based antioxidants demonstrated relatively low effectiveness due to quick elimination and low stability in the bloodstream. As one of the ways to address these issues, incorporating polymers would offer better blood stability, bioavailability, and even improved therapeutic effects [31,32,33].

**Figure 1:**
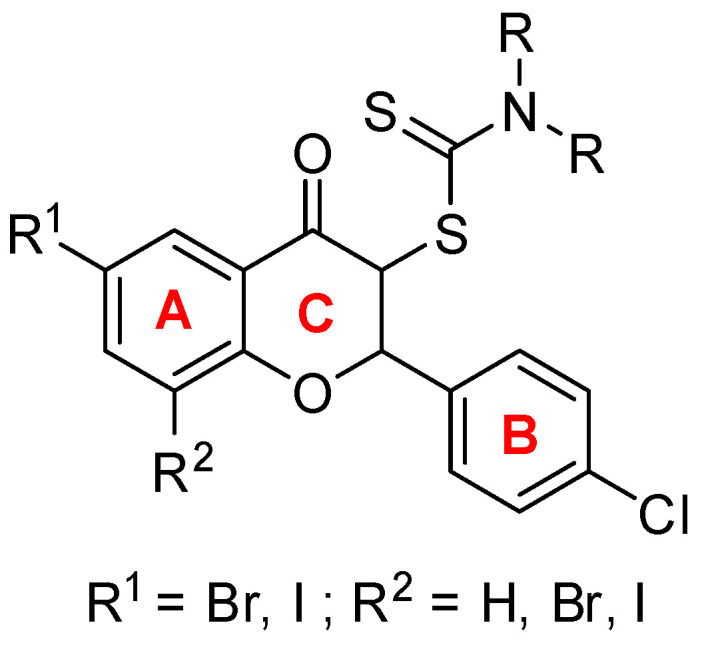
Previously investigated flavanones.

Several years ago, we disclosed a new class of synthetic flavonoids with a 1,3-dithiolium ring fused to the **C** ring of the flavan core and reported on their antimicrobial and cytotoxic properties [34,35]. Recently, we reported on the antioxidant properties of several synthetic dithiocarbamic flavanones, used as precursors in the synthesis of the above-mentioned tricyclic synthetic flavonoids (Figure 1) [36]. A structure–activity relationship study was performed in terms of influence of the substituents at the ring **A** of flavanone and of the dithiocarbamate moiety substituents (NR_2_) on the antioxidant properties of these compounds. Herein, we wish to report an extension of these studies by investigating the influence of the nature of the substituents on the flavanone ring **B** on their antioxidant properties.

## 2. Results

### 2.1. The Synthesis of Dithiocarbamic Flavanones

**Scheme 1:**
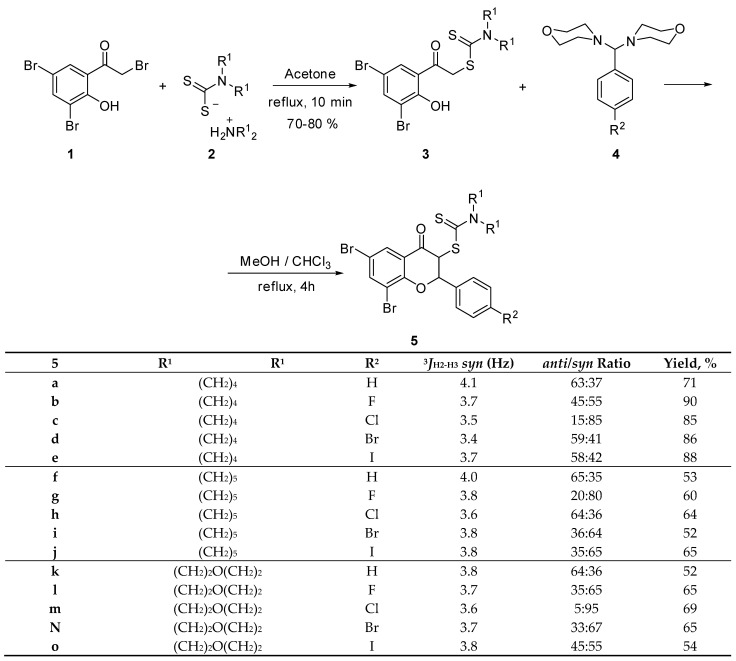
Synthesis of 3-dithiocarbamic flavanones **5a**–**o**.

**Figure 2:**
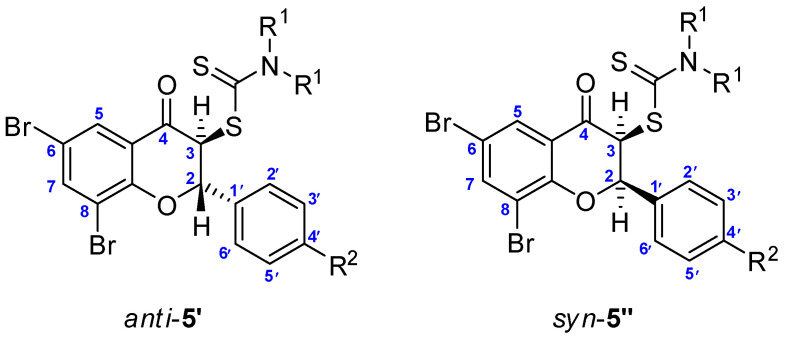
Diastereoisomers of flavonoids **5a**–**o**.

The synthetic strategy for the target 3-dithiocarbamic flavanones **5a**–**o** requires two major steps, as described in Scheme 1. The first one involves the synthesis of the corresponding phenacyl dithiocarbamates **3**. This was accomplished by the reaction of 2-bromo-1-(3,5-dibromo-2-hydroxyphenyl)ethan-1-one **1** with various salts of *N*,*N*-dialkyldithiocarbamic acid **2** according to the reported procedures in good yields (70–80%) [37,38,39]. The synthesis of the latter compounds was performed by the reaction of a secondary amine (pyrrolidine, piperidine and morpholine) with carbon disulfide [40]. In the second step, the treatment of phenacyl carbodithioates **3a**–**o** with various *para*-substituted aminals **4a**–**o** was performed using an experimental procedure reported by us, providing the expected 3-dithiocarbamic flavanones **5a**–**o** in good yields (52–90%) (Scheme 1) [41]. Thus, after 4 h in a refluxing mixture of methanol/chloroform (1:1 *v*/*v*), the desired products were isolated as non-separable mixtures of *anti*- and *syn*-isomers, as presented in Figure 2. The synthesis of flavanones **5c**, **5h** and **5m** was recently presented by us [33].

The structure of the newly synthesized 3-dithiocarbamic flavanones was proved by analytical and spectral data. The elemental analyses are presented in Supplementary Materials. The formation of the benzopyran ring was accompanied by important spectral changes. Thus, in addition to the NMR pattern of the para-substituted aromatic ring from aminal **4**, we can see the loss of the phenolic proton signal, as well as of the methylene group signal from dithiocarbamate **3** (ca. 4.8 ppm) and the appearance of the distinct pattern of hydrogen atoms near the C-2 and C-3 positions of the benzopyran ring for both diastereoisomers between 5.7 and 6.4 ppm. Since these two protons can be situated either on the same side or on opposite sides of the molecule’s plane, two diastereoisomers, *anti*-**5**′ and *syn*-**5**″, can be formed (Figure 2). The relative orientation of the two hydrogen atoms would naturally be expected to affect the size of their coupling constants. The *syn*-isomers consistently showed a coupling constant between 3.4 and 4.1 Hz, while the *anti*-isomers showed values around 7.2 Hz or appeared as multiplets due to signals overlapping.

The ^13^C NMR spectra also support the closure of the benzopyran ring. Thus, the appearance of a new signal provided by the thiocarbonyl carbon atom around 190–192 ppm and the presence of the C-2 carbon atom, found around 80.0 ppm and the C-3 carbon atom at ca. 60.0 ppm, also confirm the structure of 3-dithiocarbamic flavanones **5**. The structure of these compounds was assigned on the basis of 2D NMR studies (COSY, HMQC, HMBS) and mass spectrometric analysis.

### 2.2. Antioxidant Activities of Flavanones ***5a**–**o***

In order to investigate the antioxidant properties of the above synthesized compounds, we determined the DPPH, ABTS^+^ and FRAP scavenging activities of these. Two common antioxidants, BHT and ascorbic acid, were used as standards for the antioxidant potential comparison [42,43,44]. The experimental procedure is described in Section 4.2. The obtained results are presented in Table 1.

## 3. Discussion

As mentioned above, we previously performed a structure–activity relationship study in terms of the influence of different substituents at the ring **A** of flavanone and dithiocarbamic moiety, while keeping the same substituent (chlorine) at the para-position of ring **B** (Figure 1). [36] This study disclosed several interesting pieces of information. From the three sets of investigated dithiocarbamic flavanones, namely 6-bromo, 6,8-dibromo and 6,8-diiodo, it was found that disubstituted flavanones at ring **A** possessed higher antioxidant activities than monosubstituted ones. In fact, the latter flavanones did not exhibit better antioxidant properties than ascorbic acid and BHT. Furthermore, 6,8-dibromoflavanones were found to be better antioxidants than the corresponding 6,8-diiodo derivatives. As regards the nature of secondary amine moiety pyrrolidinyl, piperidinyl and morpholinyl derivatives were found to be the most active flavanones.

Based on these findings, we decided to undertake a study on the radical scavenging activities of three new sets of dithiocarbamic flavanones, where the 6,8-dibromo pattern at ring **A** was combined with the above-mentioned dithiocarbamic secondary amine moieties. A structure–activity relationship study was performed by varying the nature of the substituent at the para-position of ring **B** (Scheme 1). According to the obtained data presented in Table 1, the radical scavenging activities of flavanones **5a**–**o** against DPPH indicated better antioxidant activities as compared with BHT, all of these compounds displaying smaller IC_50_ values than BHT. Compound **5b** proved to be the most active with its IC_50_ value around 92 nM.

Better antioxidant activities as compared with ascorbic acid were recorded for the ABTS·+ radical scavenging of most of the flavanones **5a**–**o**. With the exceptions of **5a**, **5f**, **5i** and **5j**, all the other compounds showed smaller IC_50_ values than ascorbic acid. Special attention should be paid to flavanones **5b**,**c** and **5l**–**o**, which displayed lower IC_50_ values than those of both of the standards used, ascorbic acid and BHT. These values were in accordance with our previous findings, where pyrrolidinyl and morpholinyl derivatives exhibited the same behavior.

The FRAP radical scavenging of flavanones **5a**–**o** indicated, with four exceptions (**5a,f,j,k**), better antioxidant activity as compared with ascorbic acid. Again, pyrrolidinyl- and morpholinyl-substituted 3-dithiocarbamic flavanones presented similar antioxidant properties against both used standards. It is interesting that while all tested compounds were better antioxidants than BHT, from the above four exception, only **5j** was a *para*-halogenated flavanone.

**Scheme 2:**
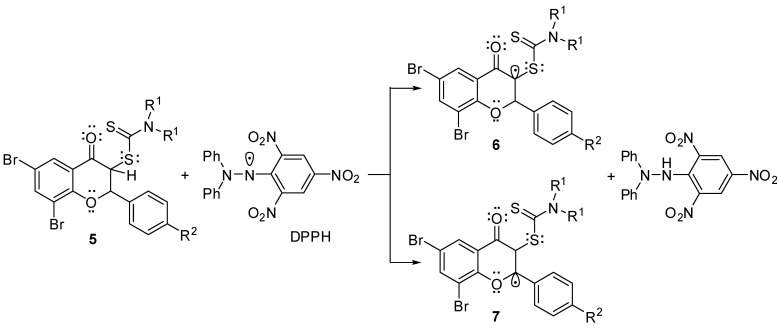
Plausible radical intermediates for the flavanone radical scavenging mechanism.

In our previous communication about antioxidant properties of 3-dithiocarbamic flavanones, we provided some insights regarding their mechanism of action [36]. We presented evidence regarding the existence of an enolate radical at the C-3 carbon atom of benzopyran ring (**6**, Scheme 2), but did not rule out the formation of a free radical at the C-2 position, which is a benzylic one (**7**, Scheme 2).

Thus, it is worthy to note that from the data presented in Table 1, an important conclusion can be drawn. In all cases, the radical scavenging activities decrease in the same order: F > Cl > Br > I > H. This order is correlated with the decrease in electronegativity and withdrawing inductive effect of these substituents. Obviously, the ease of formation of a free radical at the C-2 position is correlated with the electronic effects of the *para*-substituents at the flavanone **B** ring. These make the C(2)-H bond of the benzopyrane ring prone to hydrogen radical transfer (**7**, Scheme 2). Thus, a free hydrogen radical transfer to DPPH or ABTS·+ should provide a stable enolate radical of type **6** or a benzylic radical of type **7**. However, the presence of both free radicals at C-2 and C-3 on the same intermediate cannot be excluded.

## 4. Materials and Methods

### 4.1. Chemistry

NMR spectra were recorded on a Bruker 500 MHz spectrometer (Bruker BioSpin, Rheinstetten, Germany). Chemical shifts were reported for the major isomer in ppm downfield from TMS. Mass spectra were recorded on a Thermo Scientific ISQ LT instrument (Thermo Fisher Scientific Inc., Waltham, MA, USA). IR spectra were recorded on a Bruker Tensor 27 instrument (Bruker Optik GmbH, Ettlingen, Germany). UV-vis measurements were carried out using a Varian BioChem 100 spectrophotometer (Varian, Sydney, Australia) equipped with a 8 × 6 multicell block, thermostated with water. Melting points were obtained on a *KSPI* melting-point meter (A. KRÜSS Optronic, Hamburg, Germany) and were uncorrected. All reagents were commercially available and used without further purification. Elemental analysis (Table S1) and copies of ^13^C-NMR spectra are available in the Supplementary Materials.

#### 4.1.1. General Procedure for 6,8-Dibromo-2-phenyl-4-oxochroman-3-yl-pyrrolidine-1-carbodithioate (**5a**)

To a solution of 1-(3,5-dibromo-2-hydroxyphenyl)-1-oxoethan-2-yl-pyrrolidine-1-carbodithioate (**3a**) (0.125 g, 0.28 mmol) in a mixture of MeOH/CHCl_3_ (15 mL, 1:1 *v*/*v*), aminal **4** (0.075 g, 0.28 mmol) was added and the reaction mixture was heated under reflux for 4 h. After cooling, the solid material was filtered off and purified by recrystallization from ethanol to give **5a** (0.106 g, 71%) as colorless crystals. M.p. 165–166 °C. IR (ATR, cm^−1^) 2747, 1700, 1437, 1250, 1206, 919, 668, 410. ^1^H NMR (CDCl_3_, selected data for the major *anti*-isomer) δ 7.96 (d, ^4^*J* = 2.3 Hz, 1H), 7.91 (d, ^4^*J* = 2.3 Hz, 1H), 7.51 (m, 2H), 7.38 (m, 3H), 6.06 (d, ^3^*J* = 7.3 Hz, 1H), 5.78 (d, ^3^*J* = 7.3 Hz, 1H), 3.88 (m, 2H), 3.65 (m, 2H), 2.00 (m, 4H). ^13^C NMR (CDCl_3_, selected data for the major *anti*-isomer) δ 188.1 (C=S), 186.1 (C=O), 156.0 (C-8a), 141.5 (C-7), 134.7 (C-1′), 129.3 (C-5), 128.7 (C-3′; C-5′), 128.6 (C-2′; C-6′), 127.2 (C-4′), 122.4 (C-4a), 114.4 (C-8), 113.1 (C-6), 82.9 (C-2), 57.5 (C-3), 55.8 (C-N), 50.9 (C-N), 26.1 (CH_2_), 24.3 (CH_2_). MS (EI) *m*/*z*: 524.95 (M^+^, 4%) for C_20_H_17_^79^Br_2_NO_2_S_2_.

#### 4.1.2. 6,8-Dibromo-2-(4-fluorophenyl)-4-oxochroman-3-yl-pyrrolidine-1-carbodithioate (**5b**)

Yield 0.140 g, 90%. M.p. 174–175 °C. IR (ATR, cm^−1^) 2875, 1697, 1430, 1214, 1154, 952, 838. ^1^H NMR (CDCl_3_, selected data for the major *syn*-isomer) δ 8.04 (d, ^4^*J* = 2.3 Hz, 1H), 7.89 (d, ^4^*J* = 2.3 Hz, 1H), 7.50 (m, 2H), 7.08 (m, 2H), 6.28 (d, ^3^*J* = 3.7 Hz, 1H), 6.01 (d, ^3^*J* = 3.7 Hz, 1H), 3.87 (m, 2H), 3.66 (m, 1H), 3.60 (m, 1H), 2.03 (m, 4H). ^13^C NMR (CDCl_3_, selected data for the major *syn*-isomer) δ 188.0 (C=S), 186.5 (C=O), 163 (^1^*J_C-F_* = 248 Hz, C-4′), 155.8 (C-8a), 141.5 (C-7), 131.6 (C-1′), 129.4 (C-2′; C-6′), 128.7 (C-5), 122.2 (C-4a), 115.6 (C-3′; C-5′), 114.7 (C-8), 113.2 (C-6), 80.8 (C-2), 56.9 (C-3), 56.2 (C-N), 50.9 (C-N), 26.1 (CH_2_), 24.1 (CH_2_). MS (EI) *m*/*z*: 542.86 (M^+^, 2%) for C_20_H_16_^79^Br_2_FNO_2_S_2_.

#### 4.1.3. 6,8-Dibromo-2-(4-bromophenyl)-4-oxochroman-3-yl-pyrrolidine-1-carbodithioate (**5d**)

Yield (0.148 g, 86%). M.p. 183–184 °C. IR (ATR, cm^−1^) 2875, 1697, 1430, 1216, 1001, 808, 646, 542. ^1^H NMR (CDCl_3_, selected data for the major *anti*-isomer) δ 7.98 (d, ^4^*J* = 2.1 Hz, 1H), 7.91 (d, ^4^*J* = 2.1 Hz, 1H), 7.52 (d, ^3^*J* = 8.3 Hz, 2H), 7.41 (d, ^3^*J* = 8.3 Hz, 2H), 5.96 (m, 1H), 5.76 (m, 1H), 3.87 (m, 2H), 3.66 (m, 2H), 2.01 (m, 4H). ^13^C NMR (CDCl_3_, selected data for the major *anti*-isomer) δ 187.8 (C=S), 185.8 (C=O), 155.8 (C-8a), 141.6 (C-7), 134.8 (C-1′), 131.8 (C-3′; C-5′), 129.1 (C-2′; C-6′), 128.4 (C-5), 123.3 (C-4a), 123.0 (C-4′), 114.6 (C-8), 113.1 (C-6), 82.4 (C-2), 57.7 (C-3), 55.9 (C-N), 50.9 (C-N), 26.1 (CH_2_), 24.3 (CH_2_). MS (EI) *m*/*z*: 604.84 (M^+^, 1%) for C_20_H_16_^79^Br_3_NO_2_S_2_.

#### 4.1.4. 6,8-Dibromo-2-(4-iodophenyl)-4-oxochroman-3-yl-pyrrolidine-1-carbodithioate (**5e**)

Yield (0.163 g, 88%). M.p. 188–189 °C. IR (ATR, cm^−1^) 2883, 1693, 1430, 1260, 1002, 800, 537, 435. ^1^H NMR (CDCl_3_, selected data for the major *anti*-isomer) δ 7.97 (d, ^4^*J* = 2.4 Hz, 1H), 7.91 (d, ^4^*J* = 2.4 Hz, 1H), 7.72 (d, ^3^*J* = 8.2 Hz, 2H), 7.26 (d, ^3^*J* = 8.2 Hz, 2H), 5.94 (m, 1H), 5.75 (m, 1H), 3.88 (m, 2H), 3.66 (m, 1H), 3.61 (m, 1H), 2.01 (m, 4H). ^13^C NMR (CDCl_3_, selected data for the major *anti*-isomer) δ 187.8 (C=S), 185.8 (C=O), 155.8 (C-8a), 141.6 (C-7), 137.8 (C-3′; C-5′), 135.5 (C-1′), 129.3 (C-2′; C-6′), 128.6 (C-5), 122.2 (C-4a), 114.6 (C-8), 113.1 (C-6), 95.2 (C-4′), 82.6 (C-2), 57.6 (C-3), 55.9 (C-N), 50.9 (C-N), 26.1 (CH_2_), 24.3 (CH_2_). MS (EI) *m*/*z*: 650.82 (M^+^, 1%) for C_20_H_16_^79^Br_2_INO_2_S_2_.

#### 4.1.5. 6,8-Dibromo-2-phenyl-4-oxochroman-3-yl-piperidine-1-carbodithioate (**5f**)

Yield (0.250 g, 53%). M.p. 169–170 °C. IR (ATR, cm^−1^) 2855, 1694, 1444, 1265, 765, 697, 651, 543. ^1^H NMR (CDCl_3_, selected data for the major *anti*-isomer) δ 7.96 (d, ^4^*J* = 2.1 Hz, 1H), 7.91 (d, ^4^*J* = 2.1 Hz, 1H), 7.50 (m, 2H), 7.37 (m, 3H), 6.07 (d, *J* = 7.1 Hz, 1H), 5.82 (d, *J* = 7.1 Hz, 1H), 4.23 (m, 2H), 3.84 (m, 2H), 1.71 (m, 6H). ^13^C NMR (CDCl_3_, selected data for the major *anti*-isomer) δ 190.9 (C=S), 186.1 (C=O), 155.9 (C-8a), 141.5 (C-7), 135.7 (C-1′), 129.3 (C-5), 128.9 (C-4′), 128.6 (C-3′; C-5′), 127.2 (C-2′; C-6′), 123.2 (C-4a), 114.3 (C-8), 113.1 (C-6), 83.1 (C-2), 58.1 (C-3), 54.8 (C-N), 51.2 (C-N), 26.1 (CH_2_), 25.3 (CH_2_), 24.1 (CH_2_). MS (EI) *m*/*z*: 538.92 (M^+^, 3%) for C_21_H_19_^79^Br_2_NO_2_S_2_.

#### 4.1.6. 6,8-Dibromo-2-(4-fluorophenyl)-4-oxochroman-3-yl-piperidine-1-carbodithioate (**5g**)

Yield (0.286 g, 60%). M.p. 178–179 °C. IR (ATR, cm^−1^) 2850, 1693, 1509, 1448, 1217, 840, 718, 652. ^1^H NMR (CDCl_3_, selected data for the major *syn*-isomer) δ 8.03 (d, ^4^*J* = 1.9 Hz, 1H), 7.89 (d, ^4^*J* = 1.9 Hz, 1H), 7.49 (m, 2H), 7.07 (m, 2H), 6.35 (d, ^3^*J* = 3.8 Hz, 1H), 6.01 (d, ^3^*J* = 3.8 Hz, 1H), 4.25 (m, 2H), 3.84 (m, 2H), 1.69 (m, 6H). ^13^C NMR (CDCl_3_, selected data for the major *syn*-isomer) δ 190.4 (C=S), 186.5 (C=O), 162.8 (^1^*J_C–F_* = 248 Hz, C-4′), 155.5 (C-8a), 141.6 (C-7), 130.6 (C-1′), 129.5 (C-2′; C-6′), 128.9 (C-5), 122.4 (C-4a), 115.6 (C-3′; C-5′), 114.6 (C-8), 113.2 (C-6), 80.9 (C-2), 57.6 (C-3), 54.8 (C-N), 51.8 (C-N), 26.1 (CH_2_), 25.4 (CH_2_), 24.1 (CH_2_). MS (EI) *m*/*z*: 556.92 (M^+^, 1%) for C_21_H_18_^79^Br_2_FNO_2_S_2_.

#### 4.1.7. 6,8-Dibromo-2-(4-bromophenyl)-4-oxochroman-3-yl-piperidine-1-carbodithioate (**5i**)

Yield (0.25 g, 52%). M.p. 191–192 °C. IR (ATR, cm^−1^) 2859, 1693, 1449, 1266, 1233, 809, 647, 543. ^1^H NMR (CDCl_3_, selected data for the major *syn*-isomer) δ 8.03 (d, ^4^*J* = 2.3 Hz, 1H), 7.90 (d, ^4^*J* = 2.3 Hz, 1H), 7.50 (m, 2H), 7.40 (m, 2H), 6.35 (d, ^3^*J* = 3.8 Hz, 1H), 5.98 (d, ^3^*J* = 3.8 Hz, 1H), 4.22 (m, 2H), 3.84 (m, 2H), 1.69 (m, 6H). ^13^C NMR (CDCl_3_, selected data for the major *syn*-isomer) δ 190.3 (C=S), 186.4 (C=O), 155.4 (C-8a), 141.5 (C-7), 133.8 (C-1′), 131.6 (C-3′; C-5′), 129.5 (C-5), 128.6 (C-2′; C-6′), 123.0 (C-4a), 122.3 (C-4′), 114.7 (C-8), 113.2 (C-6), 80.9 (C-2), 57.3 (C-3), 54.2 (C-N), 51.9 (C-N), 26.1 (CH_2_), 25.3 (CH_2_), 24.1 (CH_2_). MS (EI) *m*/*z*: 616.84 (M^+^, 1%) for C_21_H_18_^79^Br_3_NO_2_S_2_.

#### 4.1.8. 6,8-Dibromo-2-(4-iodophenyl)-4-oxochroman-3-yl-piperidine-1-carbodithioate (**5j**)

Yield (0.275 g, 65%). M.p. 198–199 °C. IR (ATR, cm^−1^) 2941, 1693, 1484, 1266, 806, 544, 471. ^1^H NMR (CDCl_3_, selected data for the major *syn*-isomer) δ 8.03 (d, ^4^*J* = 2.3 Hz, 1H), 7.89 (d, ^4^*J* = 2.3 Hz, 1H), 7.71 (m, 2H), 7.27 (m, 2H), 6.35 (d, ^3^*J* = 3.8 Hz, 1H), 5.97 (d, ^3^*J* = 3.8 Hz, 1H), 4.23 (m, 2H), 3.85 (m, 2H), 1.67 (m, 6H). ^13^C NMR (CDCl_3_, selected data for the major *syn*-isomer) δ 190.6 (C=S), 185.9 (C=O), 155.8 (C-8a), 141.5 (C-7), 137.7 (C-3′; C-5′), 135.4 (C-1′), 129.3 (C-2′; C-6′), 128.8 (C-5), 122.3 (C-4a), 114.6 (C-8), 113.1 (C-6), 95.1 (C-4′), 81.0 (C-2), 57.3 (C-3), 54.2 (C-N), 52.1 (C-N), 26.0 (CH_2_), 25.3 (CH_2_), 24.1 (CH_2_). MS (EI) *m*/*z*: 664.81 (M^+^, 1%) for C_21_H_18_^79^Br_2_INO_2_S_2_.

#### 4.1.9. 6,8-Dibromo-2-phenyl-4-oxochroman-3-yl-morpholine-4-carbodithioate (**5k**)

Yield (0.159 g, 52%). IR (ATR, cm^−1^) 2910, 1708, 1584, 1449, 1261, 1225, 810, 640, 544. ^1^H NMR (CDCl_3_, selected data for the major *anti*-isomer) δ 7.97 (d, ^4^*J* = 2.2 Hz, 1H), 7.91 (d, ^4^*J* = 2.2 Hz, 1H), 7.50 (m, 2H), 7.39 (m, 3H), 6.02 (m, 1H), 5.83 (m, 1H), 4.28 (m, 2H), 3.91 (m, 2H), 3.73 (m, 4H). ^13^C NMR (CDCl_3_, selected data for the major *anti*-isomer) δ 192.8 (C=S), 185.8 (C=O), 155.9 (C-8a), 141.6 (C-7), 135.5 (C-1′), 129.3 (C-5), 129.1 (C-4′), 128.7 (C-3′; C-5′), 127.3 (C-2′; C-6′), 123.0 (C-4a), 114.5 (C-8), 113.1 (C-6), 82.9 (C-2), 66.1 (C-O), 66.0 (C-O), 58.0 (C-3), 52.2 (C-N), 50.9 (C-N). MS (EI) *m*/*z*: 540.91 (M^+^, 2%) for C_20_H_17_^79^Br_2_NO_3_S_2_.

#### 4.1.10. 6,8-Dibromo-2-(4-fluorophenyl)-4-oxochroman-3-yl-morpholine-4-carbodithioate (**5l**)

(0.185 g, 65%). M.p. 173–174 °C. IR (ATR, cm^−1^) 2908, 1704, 1581, 1446, 1268, 1227, 805, 632, 538. ^1^H NMR (CDCl_3_, selected data for the major *syn*-isomer) δ 8.06 (d, ^4^*J* = 2.3 Hz, 1H), 7.92 (d, ^4^*J* = 2.3 Hz, 1H), 7.49 (m, 2H), 7.06 (m, 2H), 6.31 (d, ^3^*J* = 3.7 Hz, 1H), 5.94 (d, ^3^*J* = 3.7 Hz, 1H), 4.29 (m, 2H), 3.96 (m, 2H), 3.73 (m, 4H). ^13^C NMR (CDCl_3_, selected data for the major *syn*-isomer) δ 192.1 (C=S), 186.2 (C=O), 162.5 (^1^*J_C-F_* = 248 Hz, C-4′), 155.1 (C-8a), 141.4 (C-7), 134.7 (C-1′), 133.1 (C-5), 129.4 (C-3′; C-5′), 128.3 (C-2′; C-6′), 122.2 (C-4a), 115.1 (C-8), 113.4 (C-6), 80.6 (C-2), 66.4 (C-O), 66.1 (C-O), 56.9 (C-3), 51.8 (C-N), 50.6 (C-N). MS (EI) *m*/*z*: 558.88 (M^+^, 3%) for C_20_H_16_^79^Br_2_FNO_3_S_2_.

#### 4.1.11. 6,8-Dibromo-2-(4-bromophenyl)-4-oxochroman-3-yl-morpholine-4-carbodithioate (**5n**)

Yield (0.133 g, 65%). M.p. 197–198 °C. IR (ATR, cm^−1^) 2904, 1699, 1587, 1451, 1258, 1221, 817, 657, 551. ^1^H NMR (CDCl_3_, selected data for the major *syn*-isomer) δ 8.04 (d, ^4^*J* = 2.3 Hz, 1H), 7.91 (d, ^4^*J* = 2.3 Hz, 1H), 7.53 (d, ^3^*J* = 8.3 Hz, 2H), 7.39 (d, ^3^*J* = 8.3 Hz, 2H), 6.29 (d, ^3^*J* = 3.7 Hz, 1H), 5.95 (d, ^3^*J* = 3.7 Hz, 1H), 4.33 (m, 2H), 3.98 (m, 2H), 3.75 (m, 4H). ^13^C NMR (CDCl_3_, selected data for the major *syn*-isomer) δ 192.2 (C=S), 186.1 (C=O), 155.4 (C-8a), 141.6 (C-7), 133.6 (C-1′), 131.7 (C-5), 129.1 (C-3′; C-5′), 128.4 (C-2′; C-6′), 123.1 (C-4a), 122.2 (C-4′), 114.9 (C-8), 113.2 (C-6), 80.7 (C-2), 66.2 (C-O), 66.0 (C-O), 57.0 (C-3), 52.4 (C-N), 50.8 (C-N). MS (EI) *m*/*z*: 618.81 (M^+^, 1%) for C_20_H_16_^79^Br_3_NO_3_S_2_.

#### 4.1.12. 6,8-Dibromo-2-(4-iodophenyl)-4-oxochroman-3-yl-morpholine-4-carbodithioate (**5o**)

Yield (0.127 g, 54%). M.p. 207–208 °C IR (ATR, cm^−1^) 2909, 1704, 1584, 1450, 1258, 1230, 819, 6661, 555. ^1^H NMR (CDCl_3_, selected data for the major *syn*-isomer) δ 8.03 (d, ^4^*J* = 2.4 Hz, 1H), 7.90 (d, ^4^*J* = 2.4 Hz, 1H), 7.74 (d, ^3^*J* = 8.4 Hz, 2H), 7.26 (d, ^3^*J* = 8.4 Hz, 2H), 6.29 (d, ^3^*J* = 3.8 Hz, 1H), 5.97 (d, ^3^*J* = 3.8 Hz, 1H), 4.28 (m, 2H), 3.91 (m, 2H), 3.75 (m, 4H). ^13^C NMR (CDCl_3_, selected data for the major *syn*-isomer) δ 192.2 (C=S), 185.6 (C=O), 155.8 (C-8a), 141.7 (C-7), 137.8 (C-5), 129.2 (C-3′; C-5′), 128.6 (C-2′; C-6′), 122.2 (C-1′), 114.7 (C-8), 113.1 (C-6), 92.6 (C-4′), 80.8 (C-2), 66.2 (C-O), 66.1 (C-O), 58.1 (C-3), 52.3 (C-N), 50.9 (C-N). MS (EI) *m*/*z*: 666.81 (M^+^, 1%) for C_20_H_16_^79^Br_2_INO_3_S_2_.

### 4.2. In Vitro Antioxidant Activities

#### 4.2.1. 2,2-Diphenyl-1-picrylhydrazyl Radical (DPPH) Assay

The DPPH method was performed according to the reported literature procedure [45,46]. Using a standard 4 mL quartz UV cell, flavanones **5a**–**o**, at different concentrations (1.7–2.3 mM in ethanol), were added to a solution of DPPH (0.1 mM in ethanol, 2.75 mL). The flavanones were added in 50 µL batches until absorbance dropped below half of the initial value. The reaction for scavenging DPPH radicals was performed in the dark at room temperature and the absorbance was measured at 517 nm after 15 min of incubation at 37 °C. The IC_50_ (nM) was determined as the flavanones’ **5a**–**o** concentration of 50% of the DPPH scavenging capacity (Table 1).

#### 4.2.2. 2,2′-Azino-bis(3-ethylbenzothiazoline-6-sulfonic Acid) (ABTS) Assay

The ABTS assay was performed according to a slightly modified literature-reported protocol [47,48]. In short, ABTS radical cation (ABTS^+·^) was prepared by reacting an ABTS solution (7 mM) in phosphate buffer (PBS, 0.1 M, pH 7.4) with a solution of potassium persulfate (2.45 mM) in water. The mixture was left to stand at room temperature for 16 h before use. After that, the mixture was diluted with PBS to reach an absorbance value of 0.70 ± 0.5 at 734 nm. Using a standard 4 mL quartz UV cell, flavanones **5a**–**o**, at different concentrations (1.6–2.7 mM in ethanol), were added to a solution of ABTS^+·^ (2.75 mL). The flavanones were added in 10 µL batches until absorbance dropped below half of the initial value. The reaction for scavenging ABTS^+·^ radicals was performed in the dark at room temperature and the absorbance was measured at 734 nm after 15 min of incubation at 37 °C. The IC_50_ (nM) was determined as the flavanones’ **5a**–**o** concentration of 50% of the ABTS^+·^ scavenging capacity (Table 1).

#### 4.2.3. Ferric Reducing Antioxidant Power (FRAP) Assay

The FRAP method was performed according to the reported literature procedure [49]. The FRAP reagent was prepared by mixing acetate buffer (300 mM, pH 3.6), a solution of 10 mM 2,4,6-tris(2-pyridyl)triazine (TPTZ) in 40 mM HCl, and 20 mM FeCl_3_ at 10:1:1 (*v*/*v*/*v*). Using a standard 4 mL quartz UV cell, flavanones **5a**–**o**, at different concentrations (1.9–2.5 mM in ethanol), were added to a solution of FRAP (2.75 mL). The flavanones were added in 25 µL batches until absorbance dropped below half of the initial value. The reaction for scavenging radicals was performed in the dark at room temperature and the absorbance was measured at 593 nm after 20 min of incubation at 37 °C. The IC50 (nM) was determined as the flavanones’ **5a**–**o** concentration of 50% of the FRAP scavenging capacity (Table 1).

## 5. Conclusions

The antioxidant properties of selected 3-dithiocarbamic flavanone frameworks were investigated. By varying the nature of the substituent (H, F, Cl, Br and I) at the para-position of flavanone ring **B**, a structure–activity relationship study on radical scavenging activities was performed. These activities were found to decrease in the following order: F > Cl > Br > I > H, which is correlated with the decrease in electronegativity and withdrawing inductive effect of these substituents, which make the C(2)-H bond of the benzopyran ring prone to hydrogen radical transfer. For each set of analyzed flavanones, regardless of the nature of the dithiocarbamic moiety, the compounds substituted with fluorine at the para-position of the ring **B** presented the highest radical scavenging activities. This paper brings some new insights regarding the mechanism of the antioxidant activity based on the intermediate formation of benzylic and/or enolate radicals. As soon as the SAR studies indicate the most active 3-dithiocarbamic flavanones, cytotoxicity will be assessed, and their use as antioxidants will be considered. Therefore, further research is required to identify the best combination of substituents linked to flavanone rings **A** and **B** that will serve as good candidates for studying their biological activities.

## Figures and Tables

**Table 1:** Radical scavenging activities of compounds **5a**–**o** (IC_50_ values in nM) *.

R^1^	R^1^	Compound	DPPH	ABTS^+^	FRAP
(CH_2_)_4_		**5a**	179.2 ± 0.4	19.0 ± 0.6	35.2 ± 0.2
		**5b**	91.7 ± 0.5	13.5 ± 0.4	19.0 ± 0.4
		**5c**	125.9 ± 0.2	14.1 ± 0.2	20.0 ± 0.2
		**5d**	130.6 ± 0.3	15.9 ± 0.2	21.9 ± 0.3
		**5e**	132.9 ± 0.4	17.4 ± 0.4	23.7 ± 0.4
(CH_2_)_5_		**5f**	250.4 ± 0.5	22.0 ± 0.5	33.1 ± 0.5
		**5g**	215.6 ± 0.2	16.9 ± 0.2	22.1 ± 0.3
		**5h**	222.1 ± 0.4	17.4 ± 0.2	23.8 ± 0.4
		**5i**	233.7 ± 0.3	18.6 ± 0.5	25.5 ± 0.2
		**5j**	247.9 ± 0.2	21.1 ± 0.6	30.1 ± 0.4
(CH_2_)_2_O(CH_2_)_2_		**5k**	165.2 ± 0.1	14.9 ± 0.3	32.7 ± 0.3
		**5l**	139.0 ± 0.5	10.1 ± 0.2	15.6 ± 0.5
		**5m**	147.4 ± 0.2	10.7 ± 0.2	17.7 ± 0.4
		**5n**	156.0 ± 0.4	11.8 ± 0.2	20.9 ± 0.2
		**5o**	157.0 ± 0.6	12.6 ± 0.3	24.1 ± 0.3
Ascorbic acid			23.4 ± 0.2	18.0 ± 0.2	29.7 ± 0.1
BHT			265.0 ± 0.1	14.6 ± 0.3	68.1 ± 0.2

* Mean values from triplicate tests. Data are shown as the mean ± standard deviation.

## Data Availability

Data is contained within the article and Supplementary Materials.

## Supplementary Materials

The following supporting information can be downloaded at: https://www.mdpi.com/article/10.3390/ijms252413698/s1.
