# Supplementary material for: Novel Dithiocarbamic Flavanones with Antioxidant Properties—A Structure–Activity Relationship Study
Source: Int J Mol Sci. 2024 Dec 21;25(24):13698. doi: 10.3390/ijms252413698 (PMC11728272; doi:10.3390/ijms252413698)

Novel dithiocarbamic flavanones with antioxidant properties – a structure-activity relationship study

M. Lucian Birsa and Laura G. Sarbu

Supplementary Material

1. Elemental analysis **S2**

2. Copies of ^{13}C NMR spectra **S3-S13**

3. Copies of 2D NMR spectra of flavanone 5a **S14-S16**

1. Elemental analysis

Elemental analyses (C, H) were conducted using a CE440 Elemental Analyser; the results were found to be in good agreement (±0.3%) with the calculated values.

Table S1. Elemental analysis data for the newly synthesized flavanones **5**.

Compound	% C		% H	
	calcd.	found	calcd.	found
**5a**	45.56	45.74	3.25	3.18
**5b**	44.05	44.15	2.96	3.05
**5d**	39.63	39.85	2.66	2.84
**5e**	36.78	36.99	2.47	2.57
**5f**	46.59	46.81	3.54	3.38
**5g**	45.10	45.40	3.24	3.44
**5i**	40.67	40.79	2.93	3.05
**5j**	37.80	38.04	2.72	2.94
**5k**	44.21	44.36	3.15	3.08
**5l**	42.80	43.02	2.87	2.99
**5n**	38.61	38.78	2.59	2.69
**5o**	35.90	36.14	2.41	2.60

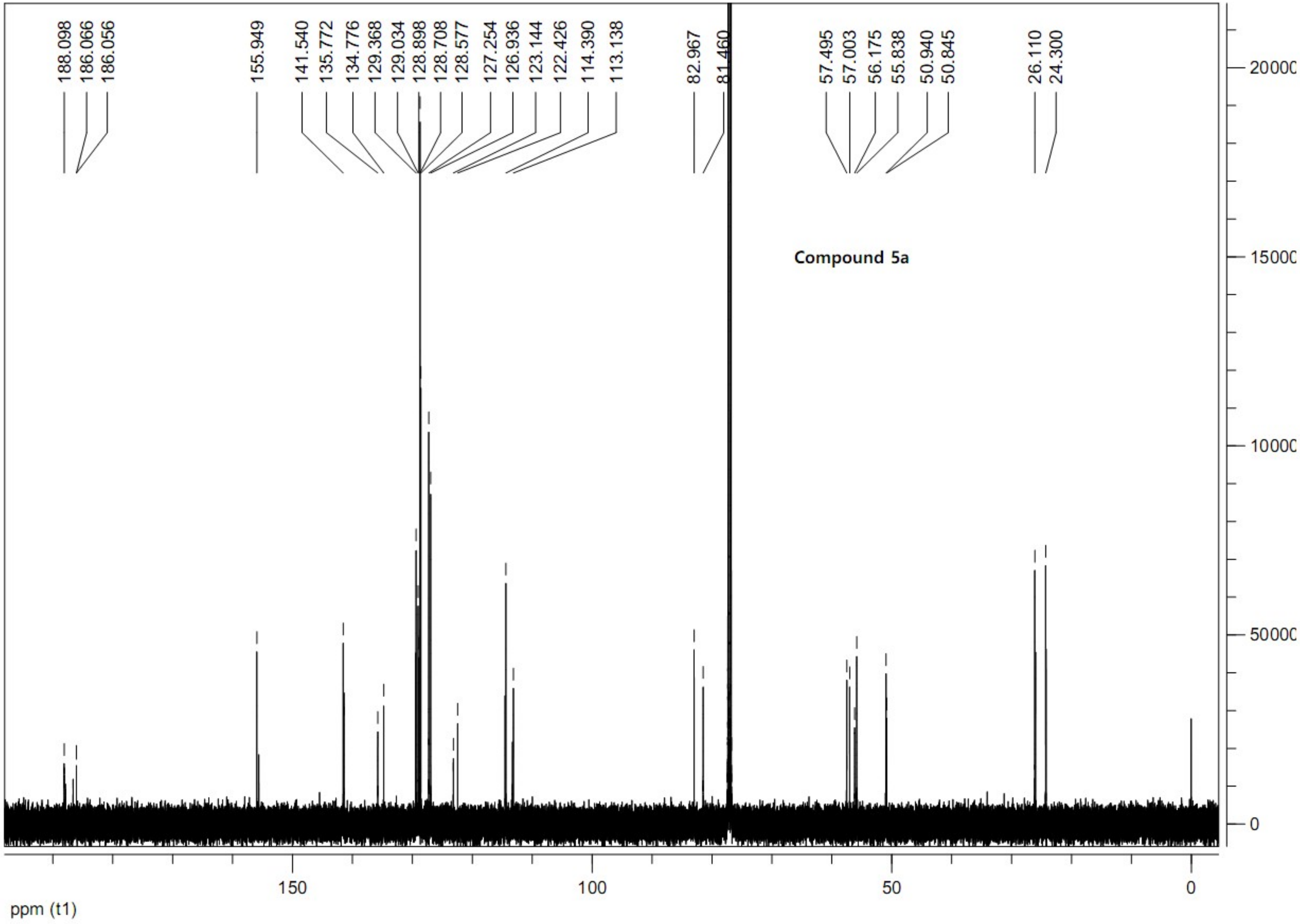
188.098
186.066
186.056
155.949
141.540
135.772
134.776
129.368
129.034
128.898
128.708
128.577
127.254
126.936
123.144
122.426
114.390
113.138
82.967
81.460
57.495
57.003
56.175
55.838
50.940
50.845
26.110
24.300
Compound 5a
20000
15000
10000
50000
0
150
100
50
0
ppm (t1)

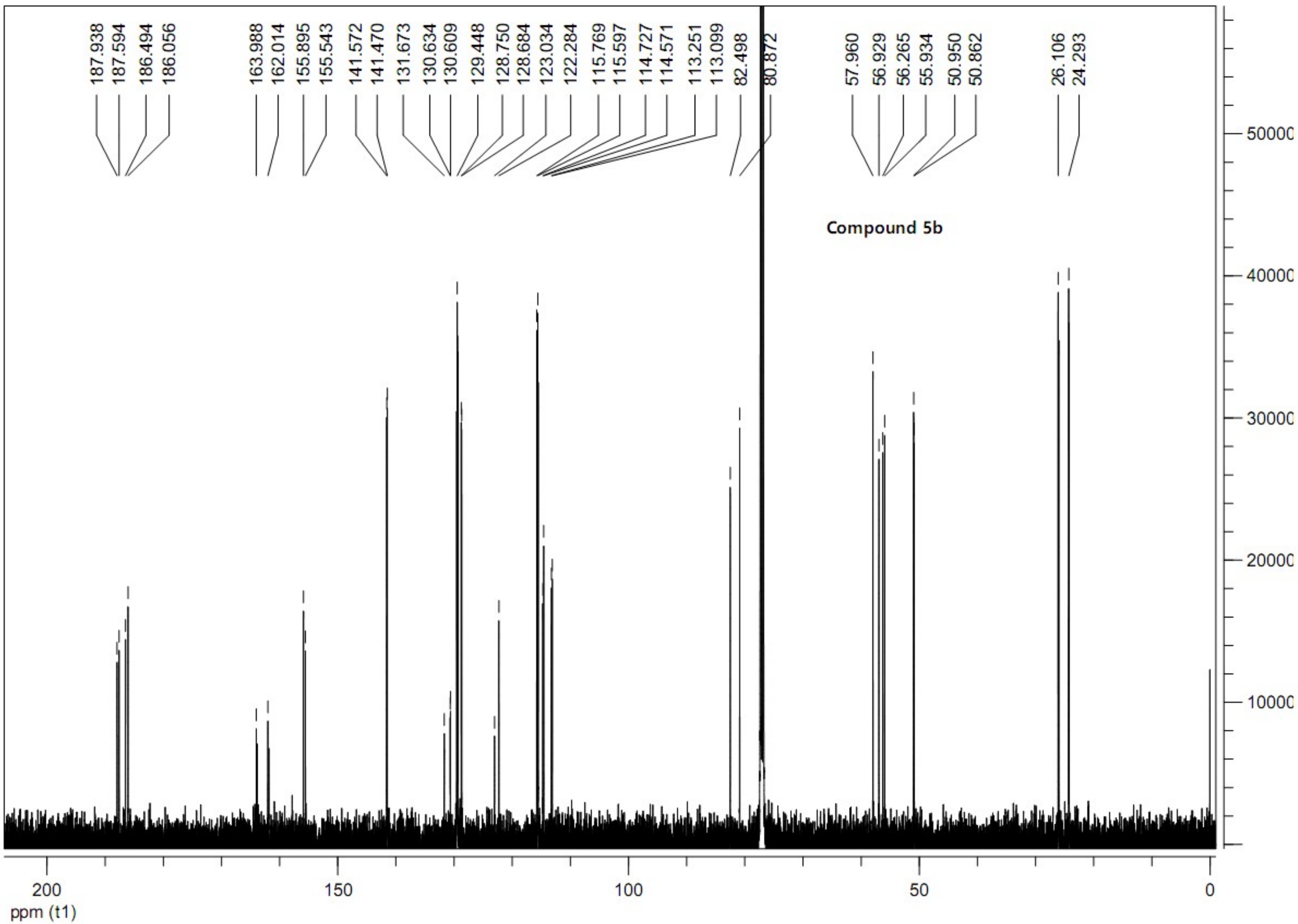
187.938
187.594
186.494
186.056
163.988
162.014
155.895
155.543
141.572
141.470
131.673
130.634
130.609
129.448
128.750
128.684
123.034
122.284
115.769
115.597
114.727
114.571
113.251
113.099
82.498
80.872
57.960
56.929
56.265
55.934
50.950
50.862
26.106
24.293
Compound 5b
50000
40000
30000
20000
10000
200
150
100
50
0
ppm (t1)

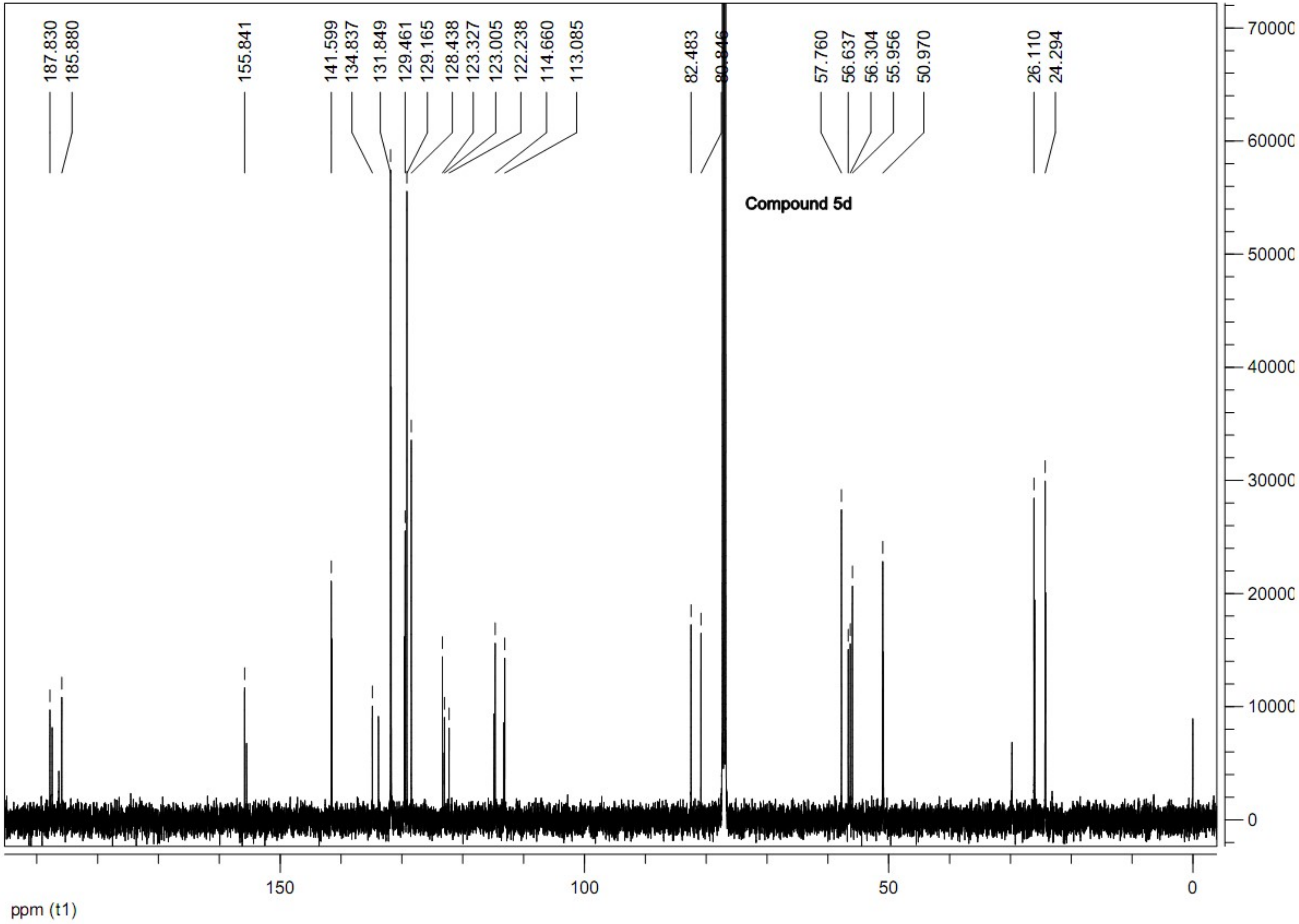
187.830
185.880
155.841
141.599
134.837
131.849
129.461
129.165
128.438
123.327
123.005
122.238
114.660
113.085
82.483
80.846
57.760
56.637
56.304
55.956
50.970
26.110
24.294
Compound 5d
70000
60000
50000
40000
30000
20000
10000
0
150
100
50
0
ppm (t1)

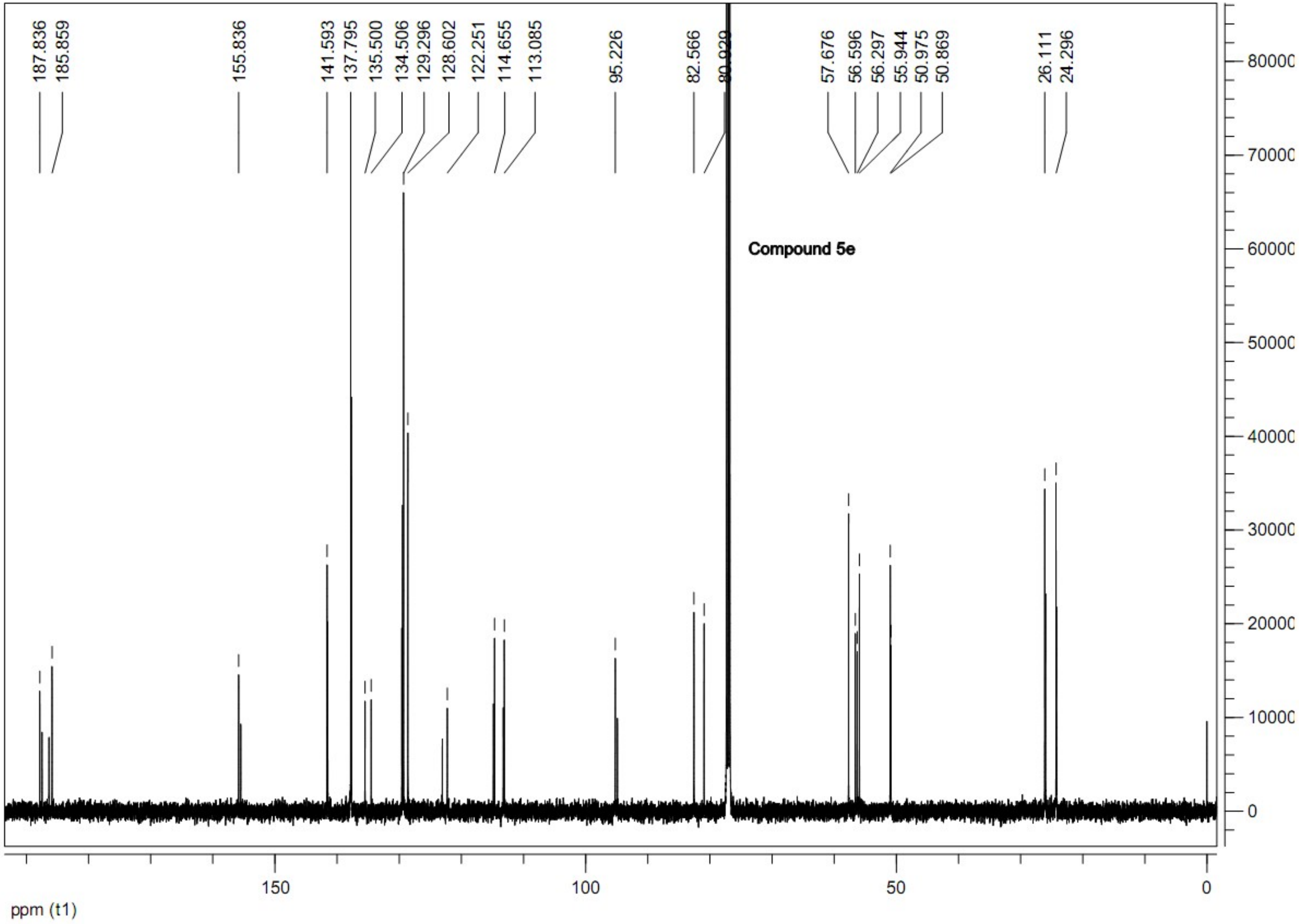
187.836
185.859
155.836
141.593
137.795
135.500
134.506
129.296
128.602
122.251
114.655
113.085
95.226
82.566
80.929
57.676
56.596
56.297
55.944
50.975
50.869
26.111
24.296
Compound 5e
80000
70000
60000
50000
40000
30000
20000
10000
0
150
100
50
0
ppm (t1)

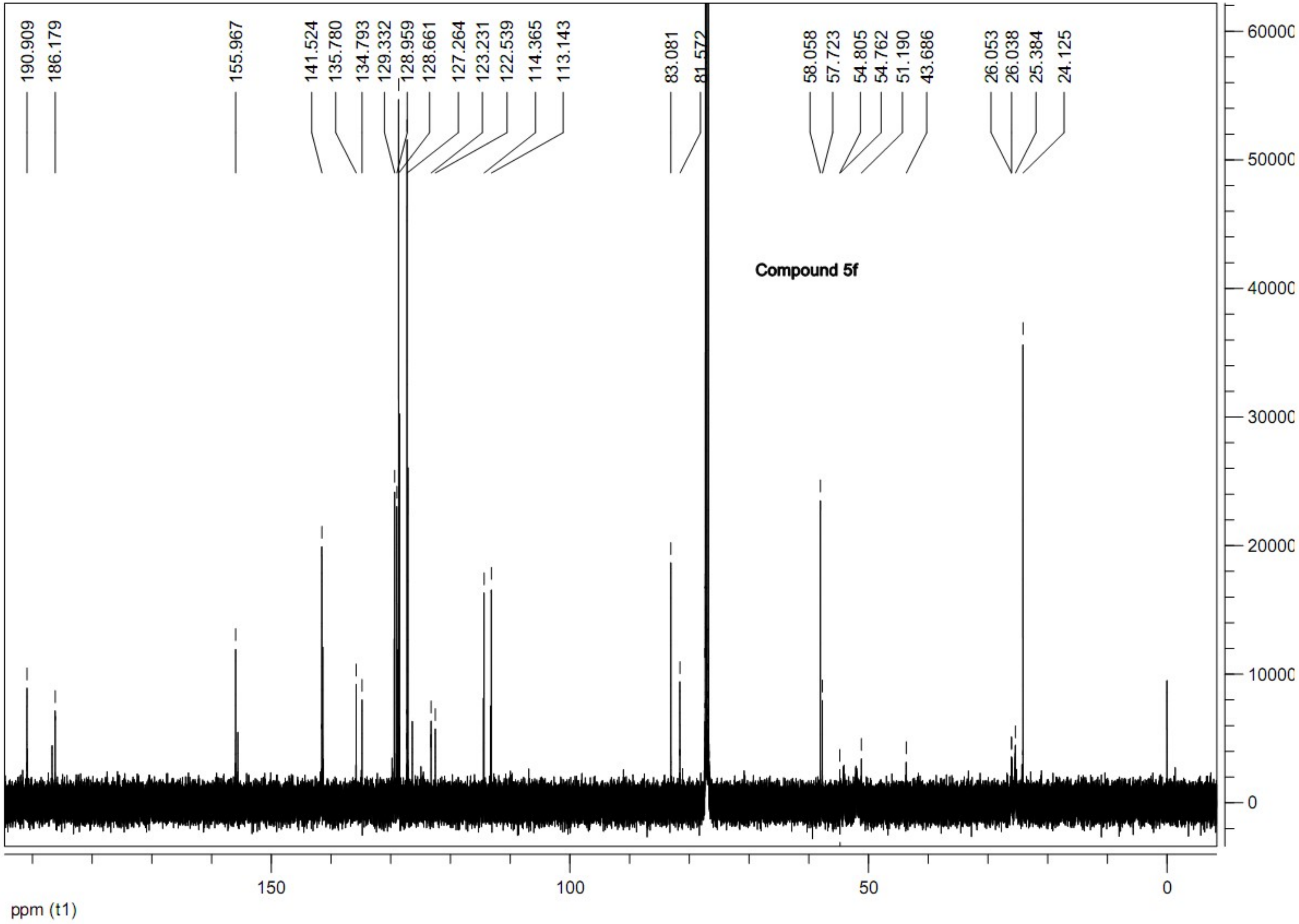
190.909
186.179
155.967
141.524
135.780
134.793
129.332
128.959
128.661
127.264
123.231
122.539
114.365
113.143
83.081
81.572
58.058
57.723
54.805
54.762
51.190
43.686
26.053
26.038
25.384
24.125
Compound 5f
60000
50000
40000
30000
20000
10000
0
150
100
50
0
ppm (t1)

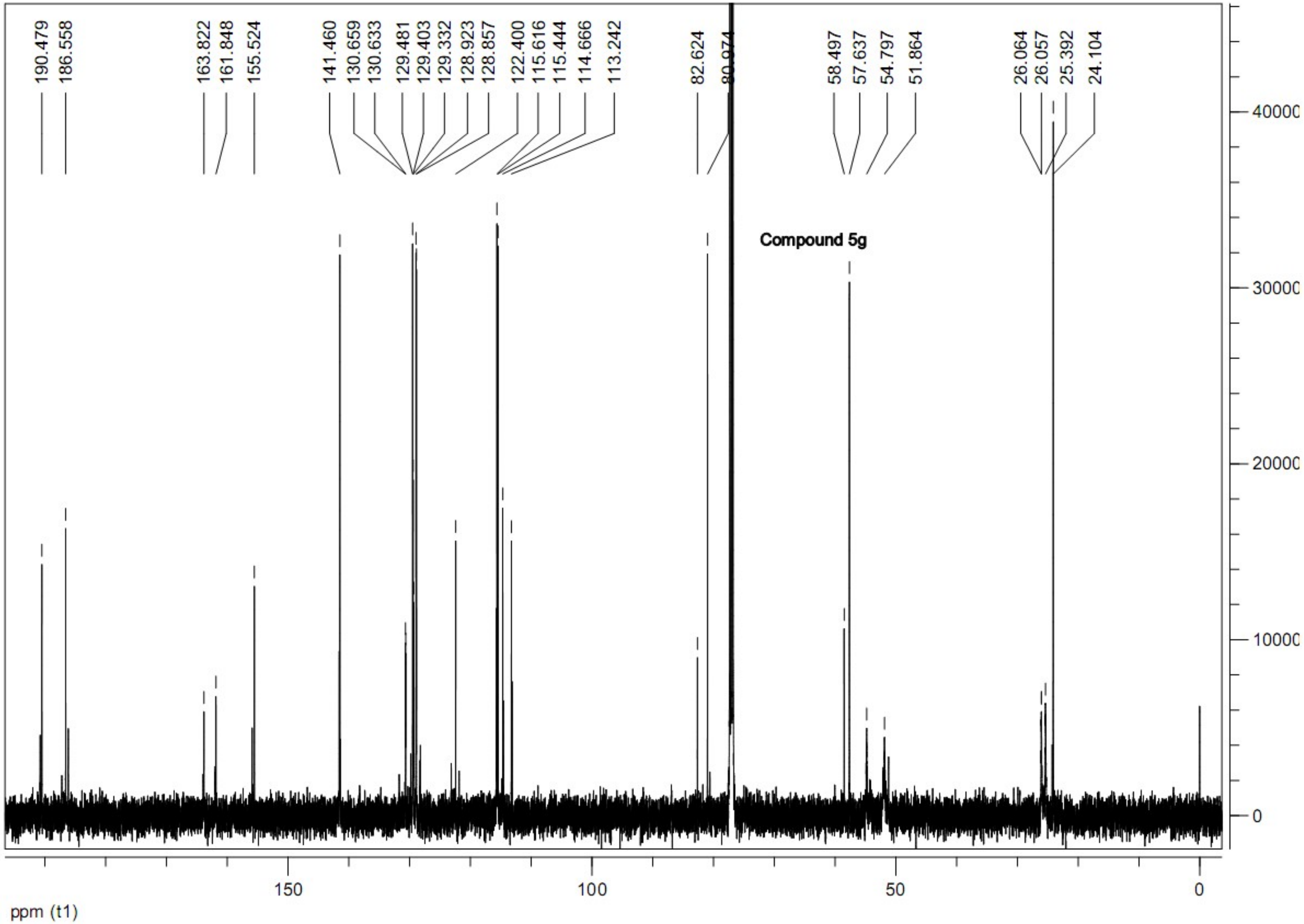
190.479
186.558
163.822
161.848
155.524
141.460
130.659
130.633
129.481
129.403
129.332
128.923
128.857
122.400
115.616
115.444
114.666
113.242
82.624
58.497
57.637
54.797
51.864
26.064
26.057
25.392
24.104
Compound 5g
40000
30000
20000
10000
0
150
100
50
0
ppm (t1)

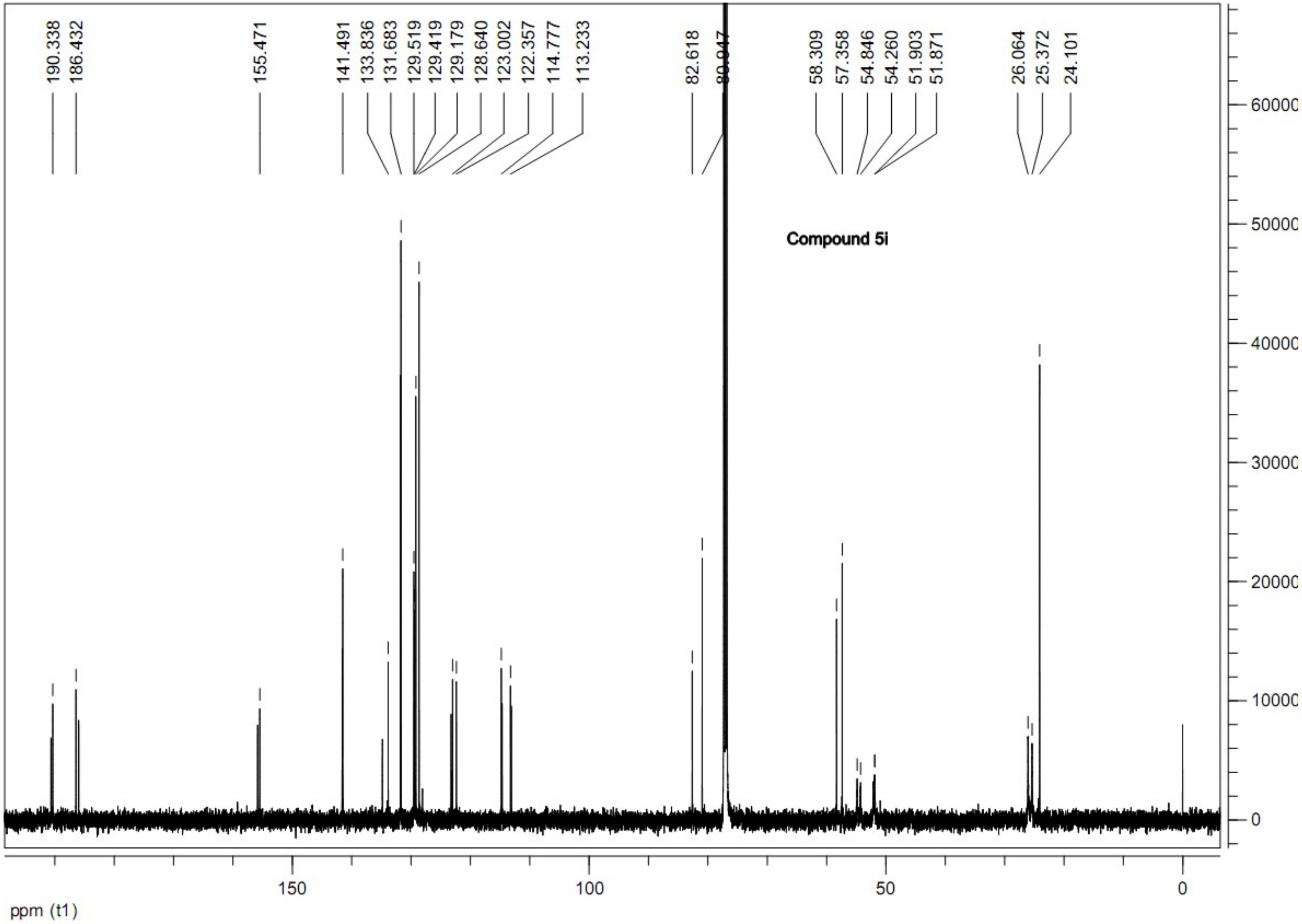
190.338
186.432
155.471
141.491
133.836
131.683
129.519
129.419
129.179
128.640
123.002
122.357
114.777
113.233
82.618
80.947
58.309
57.358
54.846
54.260
51.903
51.871
26.064
25.372
24.101
Compound 5i
60000
50000
40000
30000
20000
10000
0
150
100
50
0
ppm (t1)

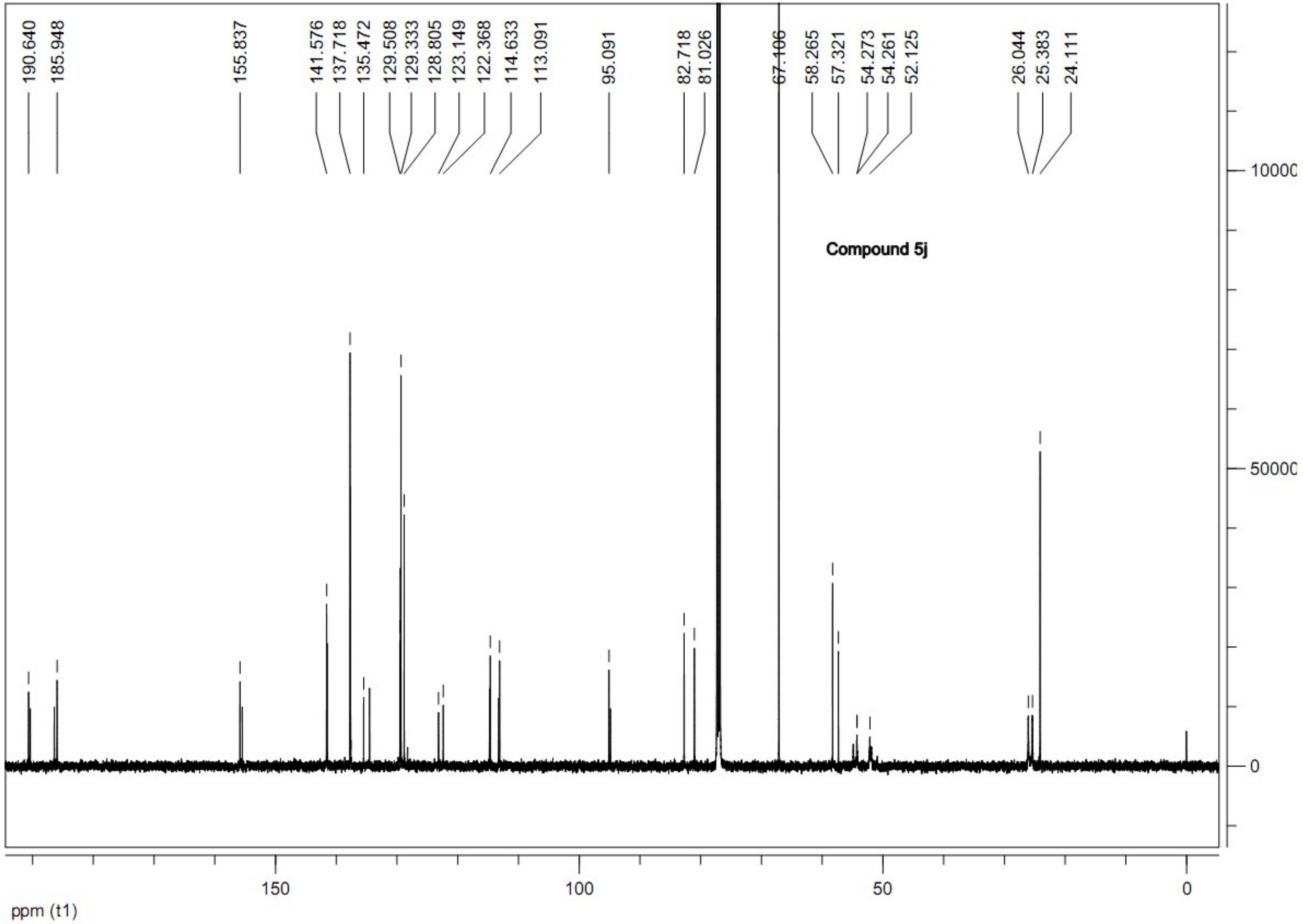

190.640
185.948
155.837
141.576
137.718
135.472
129.508
129.333
128.805
123.149
122.368
114.633
113.091
95.091
82.718
81.026
67.106
58.265
57.321
54.273
54.261
52.125
26.044
25.383
24.111
Compound 5j
10000
50000
0
150
100
50
0
ppm (t1)

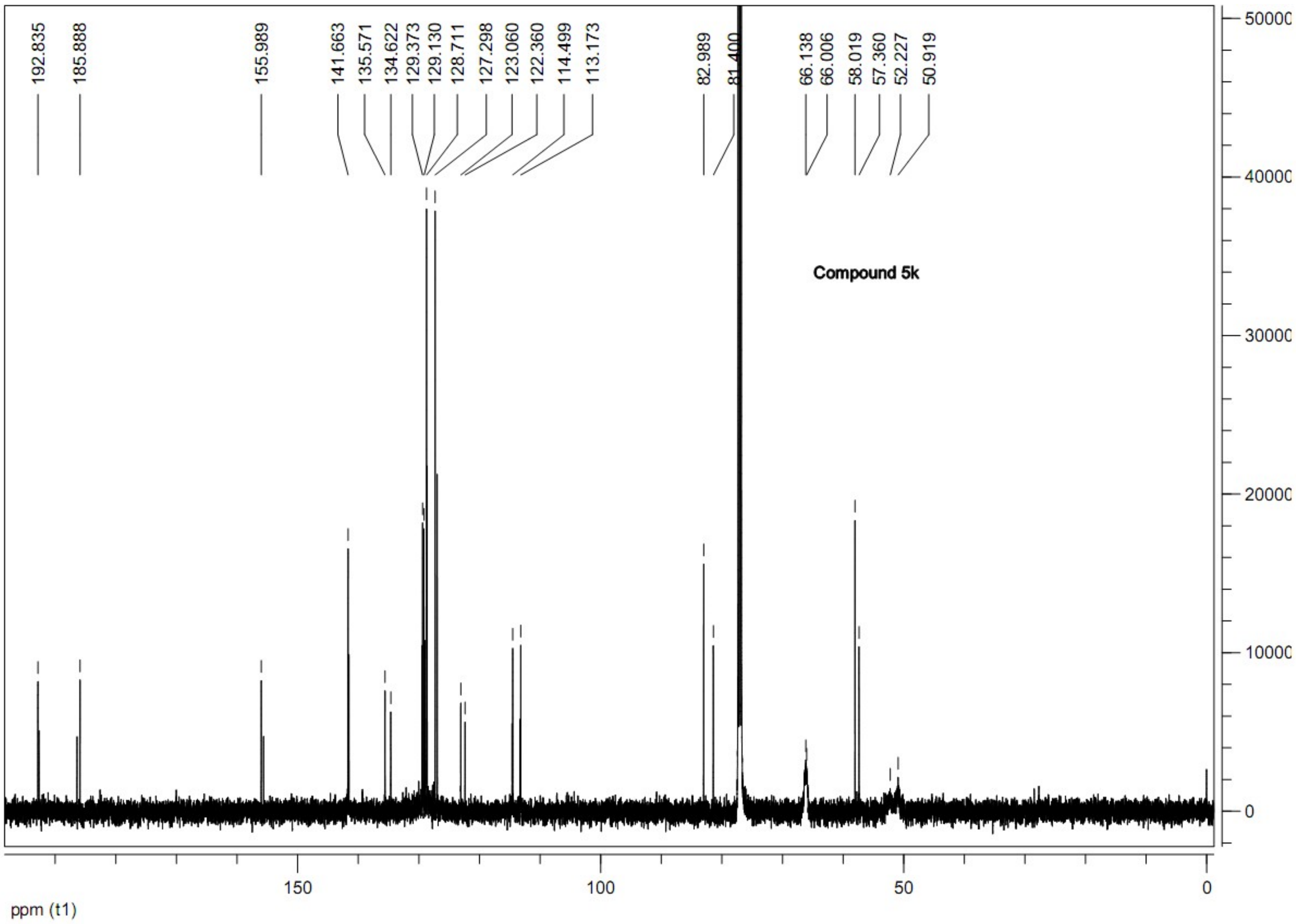
192.835
185.888
155.989
141.663
135.571
134.622
129.373
129.130
128.711
127.298
123.060
122.360
114.499
113.173
82.989
81.400
66.138
66.006
58.019
57.360
52.227
50.919
Compound 5k
50000
40000
30000
20000
10000
0
150
100
50
0
ppm (t1)

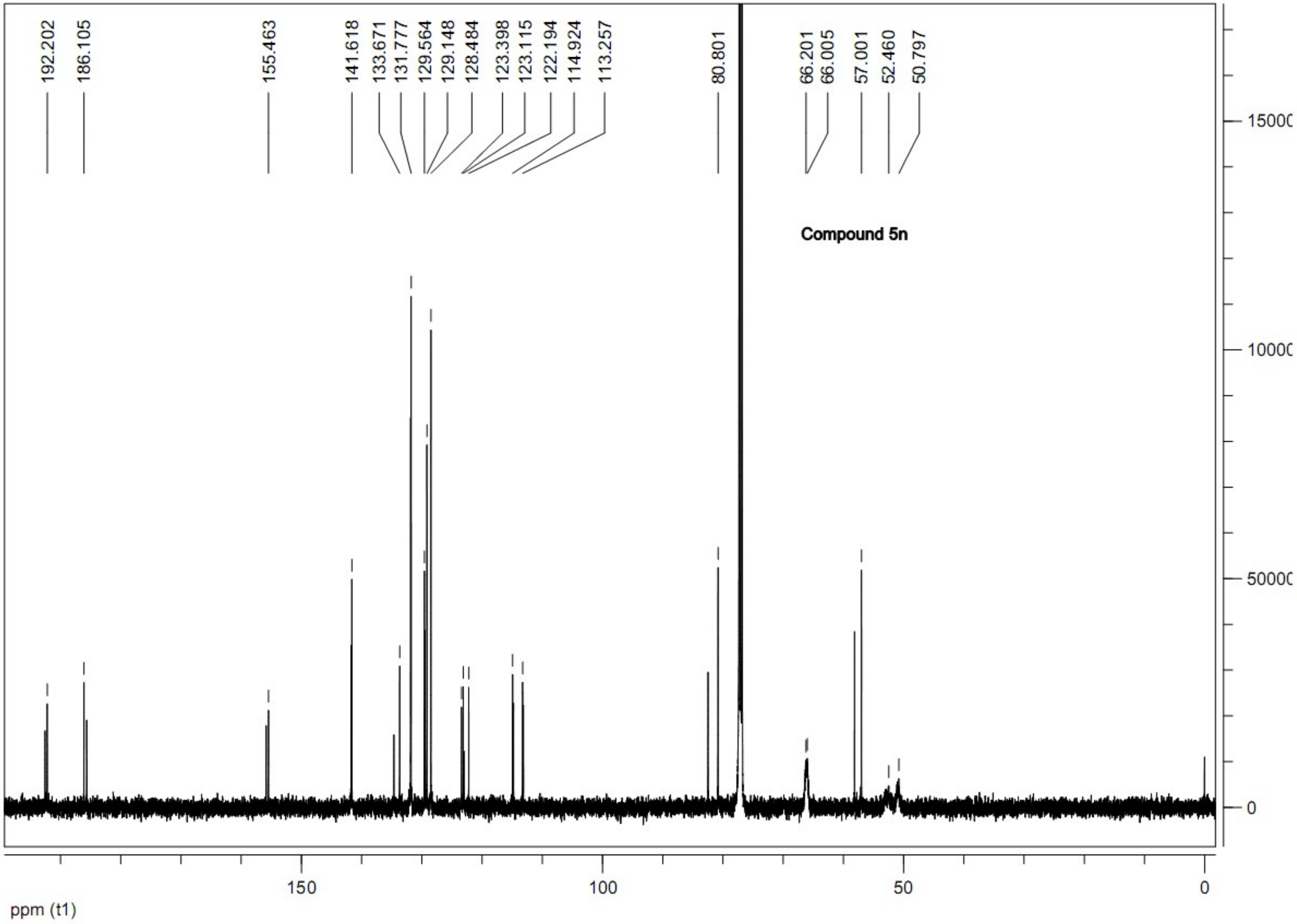
192.202
186.105
155.463
141.618
133.671
131.777
129.564
129.148
128.484
123.398
123.115
122.194
114.924
113.257
80.801
66.201
66.005
57.001
52.460
50.797
Compound 5n
15000
10000
50000
0
150
100
50
0
ppm (t1)

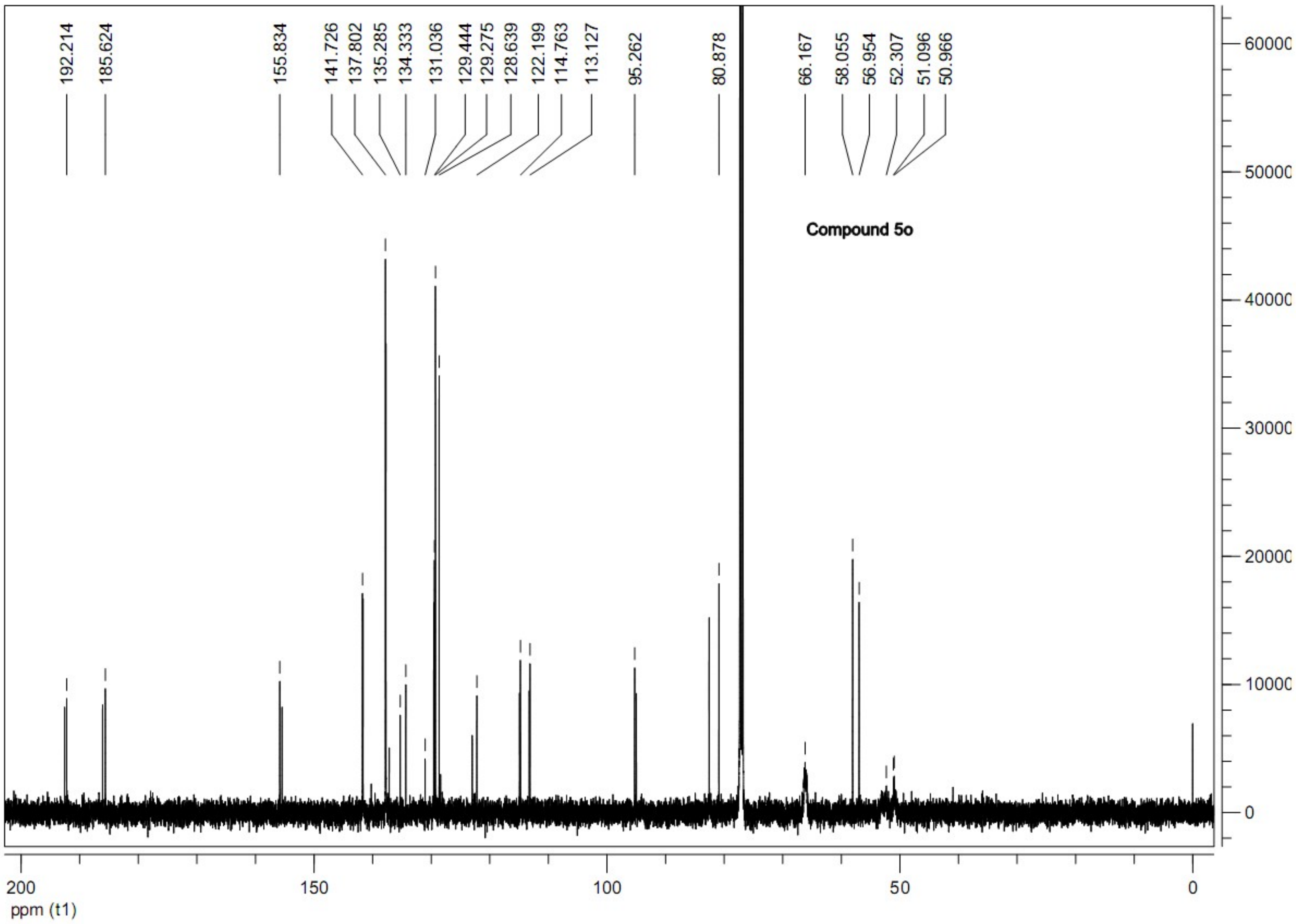

192.214
185.624
155.834
141.726
137.802
135.285
134.333
131.036
129.444
129.275
128.639
122.199
114.763
113.127
95.262
80.878
66.167
58.055
56.954
52.307
51.096
50.966
Compound 5o
60000
50000
40000
30000
20000
10000
0
200
150
100
50
0
ppm (t1)

Instrument Bruker Avance III 500 MHz UAIC
User prof. Birsa
Operator CC
Registry no. 1142
Sample Changer no. 15
Sample name 2BrPyrH-2D serie17.12.2024
@COSY45gs-BBFO-02 CDCl3
{E:\prof. Birsa\2024\dec} IconNMR-Lab 15

Current Data Parameters
NAME 241217-SC15-2BrPyrH-2DMeBrFL-OMe
EXPNO 4
PROCNO 1

F2 - Acquisition Parameters
Date_ 20241217
Time 15.21
INSTRUM spect
PROBHD 5 mm PABBO BB/
PULPROG cosygpqf45x
TD 4096
SOLVENT CDCl3
NS 1
DS 8
SWH 8012.820 Hz
FIDRES 1.956255 Hz
AQ 0.2555904 sec
RG 679.58
DW 62.400 usec
DE 6.50 usec
TE 295.5 K
D0 0.00000300 sec
D1 1.50000000 sec
D13 0.00000400 sec
D16 0.00020000 sec
IN0 0.00012500 sec

======== CHANNEL f1 ========
SFO1 500.1930011 MHz
NUC1 1H
P1 10.63 usec
PLW1 19.00000000 W

====== GRADIENT CHANNEL =====
GPNAM[1] SMSQ10.100
GPZ1 10.00 %
P16 1000.00 usec

F1 - Acquisition parameters
TD 512
SFO1 500.193 MHz
FIDRES 15.625000 Hz
SW 15.994 ppm
FnMODE QF

F2 - Processing parameters
SI 2048
SF 500.1900109 MHz
WDW QSINE
SSB 0
LB 0 Hz
GB 0
PC 1.40

F1 - Processing parameters
SI 2048
MC2 QF
SF 500.1900129 MHz
WDW QSINE
SSB 0
LB 0 Hz
GB 0

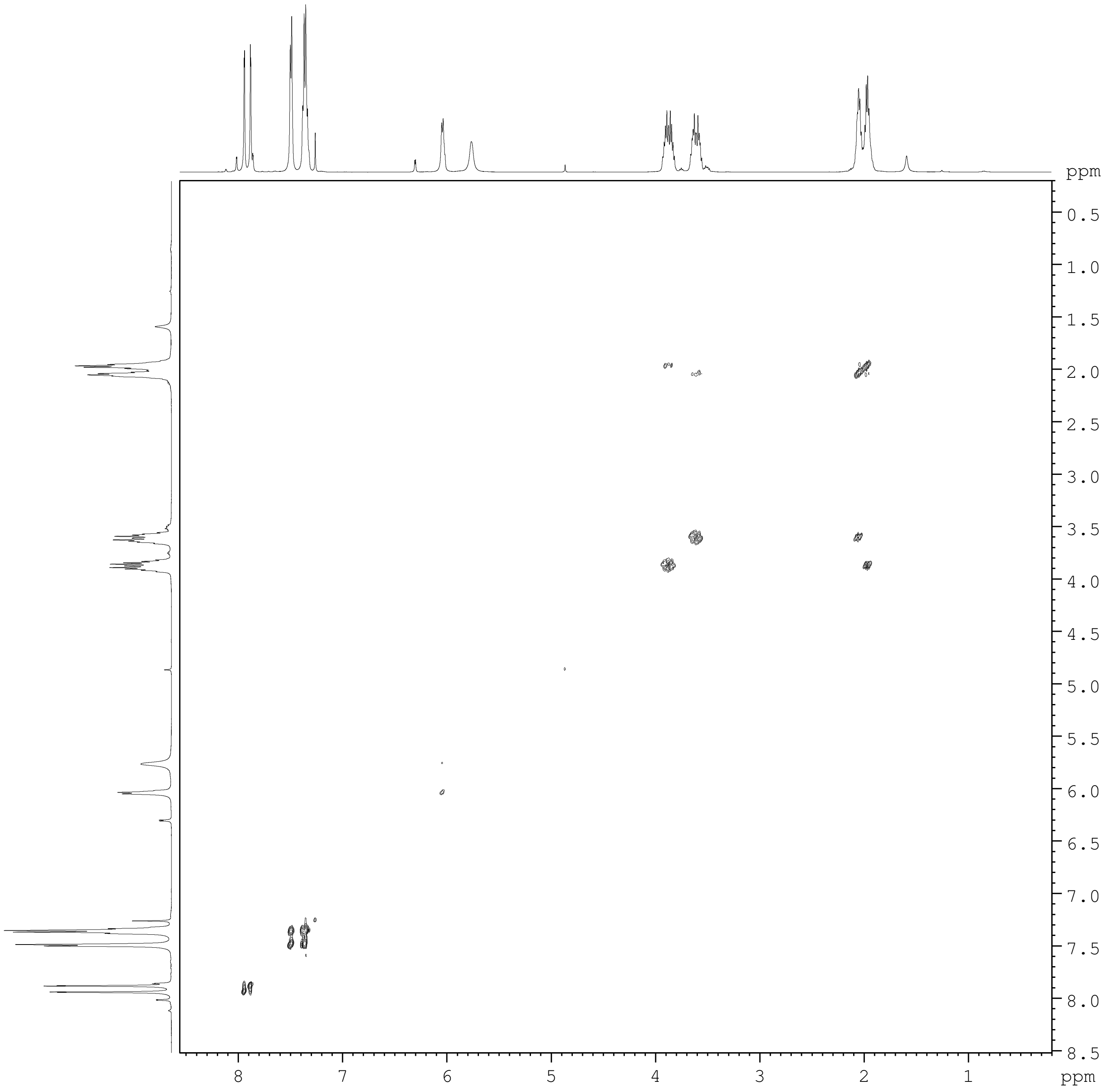

Instrument Bruker Avance III 500 MHz UAIC
User prof. Birsa
Operator CC
Registry no. 1142
Sample Changer no. 15
Sample name 2BrPyrH-2D serie17.12.2024
@HMQCgs-BBFO-02 CDCl3
{E:\prof. Birsa\2024\dec} IconNMR-Lab 15

Current Data Parameters
NAME 241217-SC15-2BrPyrH-2DMeBrFL-OMe
EXPNO 5
PROCNO 1

F2 - Acquisition Parameters
Date_ 20241217
Time 15.39
INSTRUM spect
PROBHD 5 mm PABBO BB/
PULPROG hmqcgpqf
TD 1024
SOLVENT CDCl3
NS 2
DS 16
SWH 8196.722 Hz
FIDRES 8.004611 Hz
AQ 0.0624640 sec
RG 2050
DW 61.000 usec
DE 6.50 usec
TE 295.3 K
CNST2 145.0000000
D0 0.00000300 sec
D1 1.50000000 sec
D2 0.00344828 sec
D12 0.00002000 sec
D13 0.00000400 sec
D16 0.00020000 sec
IN0 0.00001590 sec

======== CHANNEL f1 ========
SFO1 500.1935013 MHz
NUC1 1H
P1 10.63 usec
P2 21.26 usec
PLW1 19.00000000 W

======== CHANNEL f2 ========
SFO2 125.7873399 MHz
NUC2 13C
CPDPRG[2 garp
P3 9.90 usec
PCPD2 70.00 usec
PLW2 80.00000000 W
PLW12 1.60020006 W

====== GRADIENT CHANNEL =====
GPNAM[1] SMSQ10.100
GPNAM[2] SMSQ10.100
GPNAM[3] SMSQ10.100
GPZ1 50.00 %
GPZ2 30.00 %
GPZ3 40.10 %
P16 1000.00 usec

F1 - Acquisition parameters
TD 256
SFO1 125.7873 MHz
FIDRES 122.838051 Hz
SW 249.998 ppm
FnMODE QF

F2 - Processing parameters
SI 1024
SF 500.1900105 MHz
WDW QSINE
SSB 2
LB 0 Hz
GB 0
PC 1.40

F1 - Processing parameters
SI 1024
MC2 QF

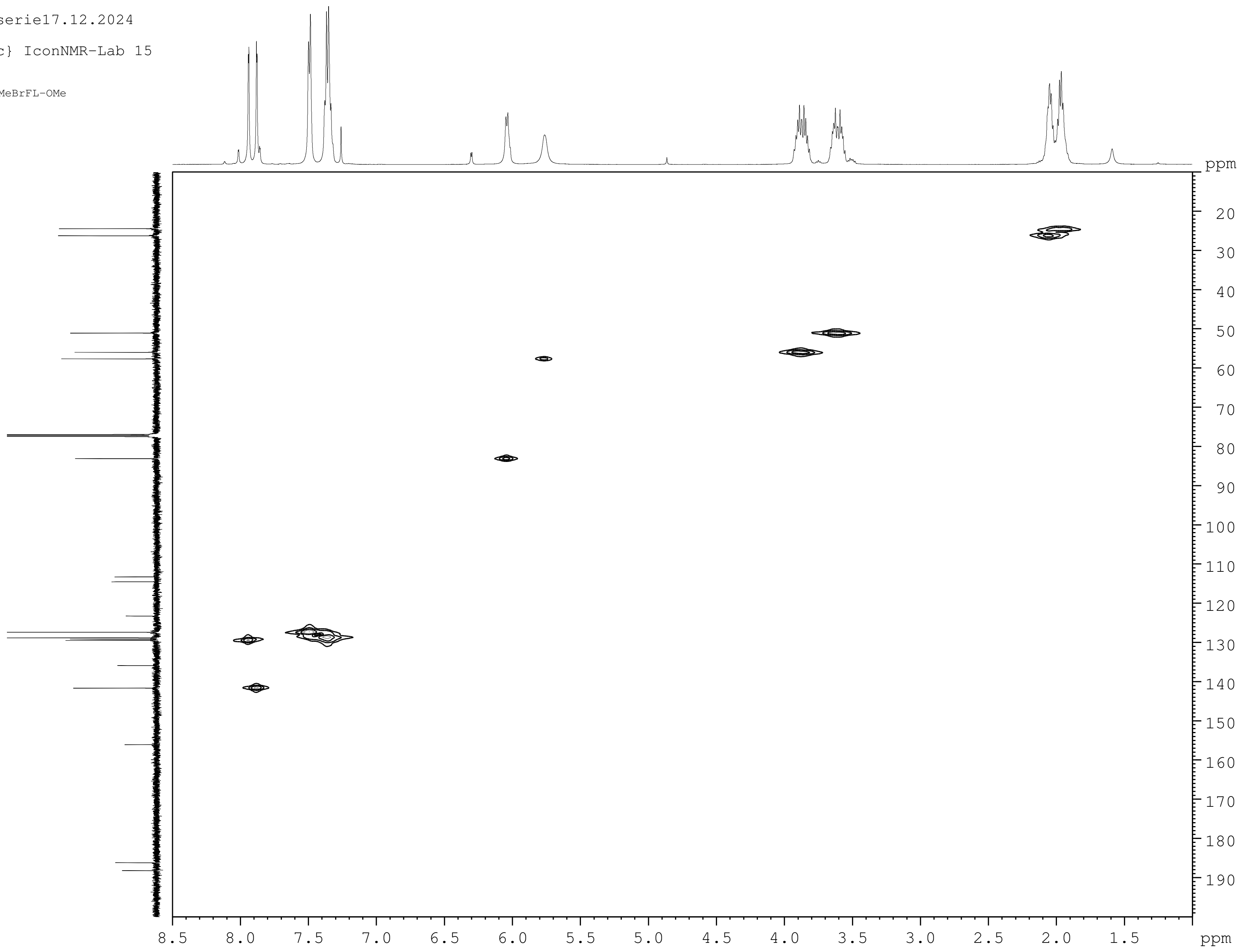

Instrument Bruker Avance III 500 MHz UAIC
User prof. Birsa
Operator CC
Registry no. 1142
Sample Changer no. 15
Sample name 2BrPyrH-2D serie17.12.2024
@HMBCgs-BBFO-02 CDCl3
{E:\prof. Birsa\2024\dec} IconNMR-Lab 15

Current Data Parameters
NAME 241217-SC15-2BrPyrH-2DMeBrFL-OMe
EXPNO 6
PROCNO 1

F2 - Acquisition Parameters
Date_ 20241217
Time 15.55
INSTRUM spect
PROBHD 5 mm PABBO BB/
PULPROG hmbcgplpndqf
TD 4096
SOLVENT CDCl3
NS 2
DS 16
SWH 8012.820 Hz
FIDRES 1.956255 Hz
AQ 0.2555904 sec
RG 2050
DW 62.400 usec
DE 6.50 usec
TE 295.4 K
CNST2 145.0000000
CNST13 10.0000000
D0 0.00000300 sec
D1 1.50000000 sec
D2 0.00344828 sec
D6 0.05000000 sec
D16 0.00020000 sec
IN0 0.00001330 sec

======== CHANNEL f1 ========
SFO1 500.1935013 MHz
NUC1 1H
P1 10.63 usec
P2 21.26 usec
PLW1 19.00000000 W

======== CHANNEL f2 ========
SFO2 125.7892265 MHz
NUC2 13C
P3 9.90 usec
PLW2 80.00000000 W

====== GRADIENT CHANNEL =====
GPNAM[1] SMSQ10.100
GPNAM[2] SMSQ10.100
GPNAM[3] SMSQ10.100
GPZ1 50.00 %
GPZ2 30.00 %
GPZ3 40.10 %
P16 1000.00 usec

F1 - Acquisition parameters
TD 512
SFO1 125.7892 MHz
FIDRES 73.425751 Hz
SW 298.865 ppm
FnMODE QF

F2 - Processing parameters
SI 4096
SF 500.1900201 MHz
WDW SINE
SSB 0
LB 0 Hz
GB 0
PC 1.40

F1 - Processing parameters
SI 2048
MC2 QF
SF 125.7728604 MHz
WDW SINE
SSB 0

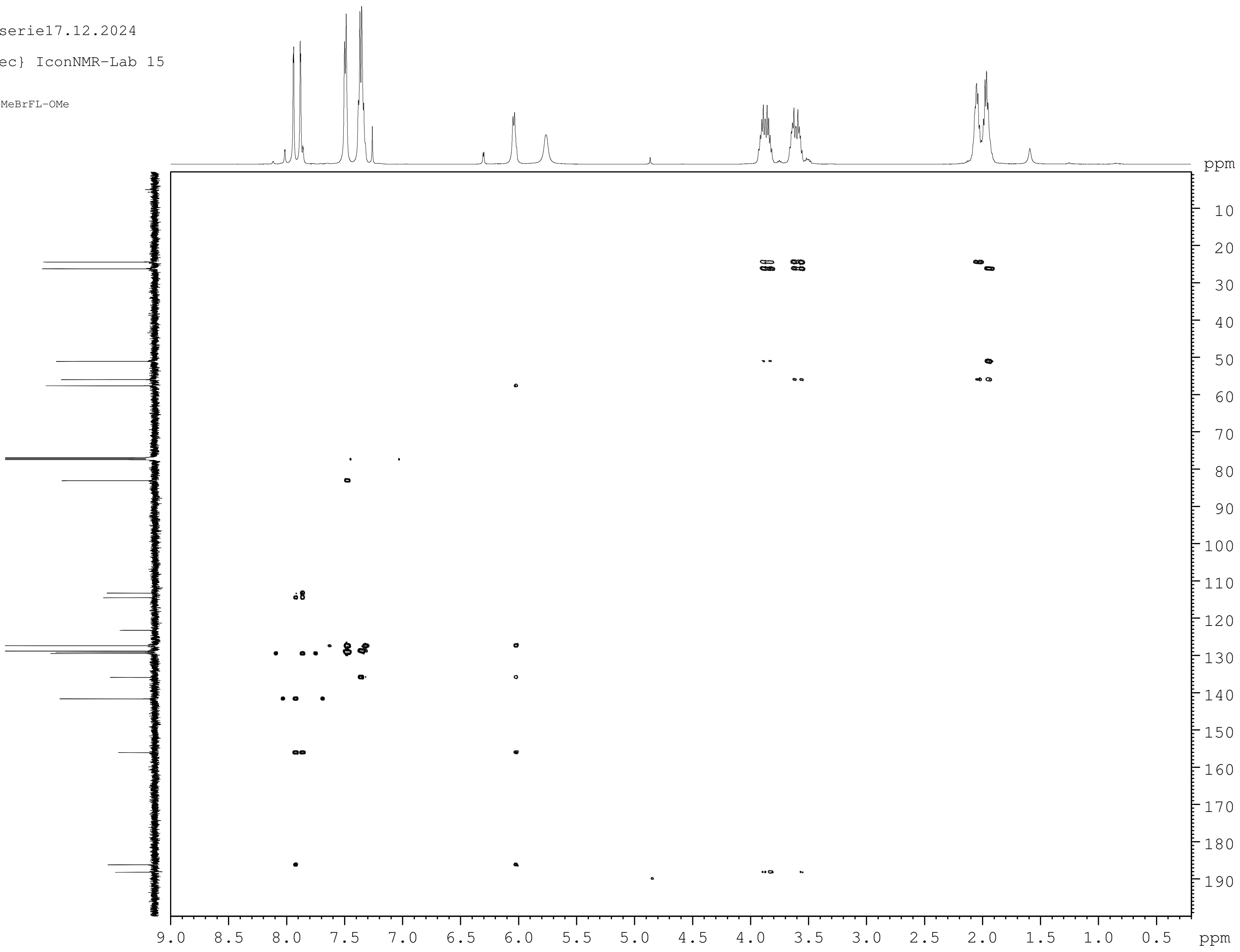

Supplement: Supplementary file 1 [file ijms-25-13698-s001.zip › ijms-3345946-supplementary.pdf]
